# Efficacy and safety of Myofascial-meridian Release Acupuncture (MMRA) for chronic neck pain: a study protocol for randomized, patient- and assessor-blinded, sham controlled trial

**DOI:** 10.1186/s12906-016-1027-y

**Published:** 2016-02-02

**Authors:** Seunghoon Lee, Dongwoo Nam, Jungtae Leem, Gajin Han, Seungmin Lee, Junhee Lee

**Affiliations:** 1 Department of Acupuncture & Moxibustion, Kyung Hee University Korean Medicine Hospital, 23 Kyungheedae-ro, Dongdaemun-gu Seoul, 02447 Republic of Korea; 2 Department of Acupuncture & Moxibustion, College of Korean Medicine, Kyung Hee University, 23 Kyungheedae-ro, Dongdaemun-gu Seoul, 02447 Republic of Korea; 3 Department of Sasang Constitutional Medicine, College of Korean Medicine, Kyung Hee University, 23 Kyungheedae-ro, Dongdaemun-gu, Seoul, 02447 Republic of Korea; 4 Korean Medicine Clinical Trial Center, Kyung Hee University Korean Medicine Hospital, 23 Kyungheedae-ro, Dongdaemun-gu, Seoul, 02447 Republic of Korea

**Keywords:** Chronic neck pain, Myofascial-meridian release acupuncture, Randomized controlled trial

## Abstract

**Background:**

The purpose of this study is to evaluate the efficacy and safety of myofascial-meridian release acupuncture (MMRA) in the treatment of chronic neck pain compared with sham acupuncture.

**Methods/design:**

A protocol for a randomized, patient- and assessor-blinded, sham controlled parallel trial is presented. Seventy-four participants with a ≥3 month history of neck pain and a score of ≥4 on the 11-point pain intensity numerical rating scale (PI-NRS) will be randomly assigned to the MMRA group (*n* = 37) or sham acupuncture group (*n* = 37). The participants will receive the MMRA treatment or sham acupuncture treatment twice per week for 4 weeks. The primary outcome is the mean change in the PI-NRS (0 = no pain and 10 = worst possible pain, 11-point Likert scale) from baseline to 4 weeks. The secondary outcomes are the mean change from baseline on the clinical relevance of the pain (ratio of changes greater than 1.5 or with percentiles greater than 30 % and 50 % in the PI-NRS), function (Neck Disability Index and Cervical Range of Motion), autonomic and psychometric measurements (Heart Rate Variability and Perceived Stress Scale), quality of life (EuroQol), global assessment (Patient Global Impression of Change), semi-objective outcomes (pressure pain threshold, consumption of rescue medicine and days of restricted activity) and immunologic/stress biomarkers. Adverse events will be evaluated at every visit.

**Discussion:**

The results of this trial will provide evidence to confirm the efficacy and safety of acupuncture for chronic neck pain.

**Trial registration:**

The trial is registered with the Clinical Research Information Service (CRiS), Republic of Korea: KCT0001573.

## Background

Neck pain is one of the most common symptoms in patients suffering from musculoskeletal pain [1, 2]. More than 70 % of adults in the general population suffer from neck pain at some point throughout their life [2, 3]. The Neck Pain Task Force reported that the 1-year prevalence of neck pain ranges from 30 to 50 % in the general population, and 2 to 11 % of these patients report restricted daily activities [4]. Neck pain is also common in most occupational groups, and 11 to 14 % of workers are restricted in their activities [4]. Not only daily life activities are diminished in chronic neck pain patients but also health-related quality of life (HRQoL). In a cross-sectional study, HRQoL was negatively correlated with prevalent neck pain [5], and the existence of neck pain was also a predictor of poor future physical HRQoL in a longitudinal study [6].

There are several modifiable risk factors, such as exposure to tobacco from direct smoking, physical activity, psychologic health and repetitive and sedentary work positions [4]. Otherwise, the pathophysiological pathway of chronic neck pain is not well established. However, there are several previous studies on the relationship between neck pain and inflammation or stress. In a human study, elevated inflammation associated chemotactic cytokines, such as IL-1β, IL-6, CRP and tumor necrosis factor-α (TNF-α), were suggested to be contributors to musculoskeletal disorders [7]. In cross-sectional results for patients with neck, shoulder and back pain, cortisol was found to be a stress biomarker and was significantly higher in the “pain” groups than in the “no pain” group [8].

Many trials have been conducted to evaluate the effect of acupuncture on chronic neck pain. However, several limitations have hindered conclusions on the value of acupuncture for chronic neck pain. First, according to a recent systematic review, many acupuncture chronic neck pain studies report subjective outcomes related to pain and disability using tools such as the visual analogue scale (VAS) and the Neck Disability Index (NDI) [9]. However, the placebo treatment had higher clinical effects than no treatment when measured by a subjective continuous outcome variable [10]. Moreover, in pain treatment, subjective outcomes cannot avoid the risk of bias [11]. Second, while there are several reviews on the effectiveness of acupuncture in chronic neck pain, no accepted mechanism is known; though, the chronic neck pain might be related with immunologic or stress factors. A lack of biological plausibility is also one of the problems of acupuncture studies about pain [12]. Third, although a minimal clinical important difference (MCID) is important in interpreting the clinical relevance of trial results, almost no trial about acupuncture for chronic neck pain considered the MCID when designing and interpreting the clinical trials. Finally, in many acupuncture trials, the treatment protocol is different from daily medical practice. Therefore, the results do not correlate well with applied clinical practice.

In view of the limitations of previous studies, our study outlines several improvements. First, we adapted a Park sham device (PSD; Acuprime, Exeter, UK) that is a widely used, validated sham device in acupuncture research [13] to evaluate the efficacy of acupuncture treatment in chronic neck pain with semi-objective outcomes, such as the pressure pain threshold, consumption of rescue medication and days of restricted activity. Second, we will explore the mechanism of acupuncture treatment in chronic neck pain and if the efficacy is mediated by inflammation and stress reduction. Third, to evaluate the clinical relevance and statistical significance, the MCID will be used to design a research protocol and interpret the results of the trial. Finally, we will reflect clinical practice by applying the myofascial-meridian release acupuncture (MMRA) treatment protocol that is used at the Spine and Joint Clinical Center in Kyung Hee University Korean Medicine Hospital, which will increase the external validity.

## Methods/design

### Objective

The aims of this study are to assess the efficacy and safety of MMRM in treating chronic neck pain for pain intensity, function, autonomic and psychometric measurements, quality of life, global assessment, biomarkers and adverse events compared with sham acupuncture.

### Design and setting

This study is an equal randomized, stratified (11-point pain intensity numerical rating scale (PI-NRS) score ranging from 4 to 6 in one group and 7 to 10 in one group), patient- and assessor-blinded, sham controlled, parallel-group, single center, clinical trial conducted in Korea.

#### Recruitment period

The participants will be recruited from the Kyung Hee University Korean Medicine Hospital in Seoul, which has a population of approximately 10,078,000. Recruitment is expected from August 2015 to April 2016.

#### Methods of recruitment

A total of 74 participants with chronic neck pain will be recruited from the outpatients of Kyung Hee University Korean Medicine Hospital. We will recruit the participants by advertising on the hospital homepage and bulletin board, in the media (local newspapers and public newsletters) and on the internet homepages of public institutions.

#### Study plan

After oral and written consent is obtained from each patient, subject information will be collected at the first visit. After a participant voluntarily consents to the study, they will be screened using inclusion/exclusion criteria at the first visit. If the participants take the pain medications, a ten-day wash-out period will be needed. At the end of the screening phase, if an eligible participant meets the study criteria with moderate (PI-NRS score range from 4 to 6) or severe (PI-NRS score range from 7 to 10) chronic neck pain, then the subject will be randomly allocated into 1 of 2 groups (the MMRA or sham acupuncture group) with a 1:1 allocation ratio. The MMRA group and sham acupuncture group will receive a total of 8 acupuncture treatment sessions during 4 weeks. MMRA acupuncture will be conducted at traditional acupuncture points, or ashi points, with deqi sensation. Sham acupuncture will be performed at non-acupuncture points without deqi sensation using a PSD. After a 4-week treatment, primary outcome measurements will be evaluated.

### Types of participants

#### Inclusion criteria

Participants who meet the following conditions will be included: (1) Males and females aged 19 to 75 years; (2) Suffering from neck pain for at least 3 months (Neck pain is defined as pain and stiffness around the cervical spine that can be aggravated with movement or pressure.); (3) Classified as Grade I, II (no radiculopathy or structural pathology) according to The Bone and Joint Decade (2000–2010) Task Force on Neck Pain; (4) A score more than 4 on PI-NRS at the time of recruitment; (5) Agreement via written informed consent after being provided with an explanation regarding the purpose and characteristics of this study.

#### Exclusion criteria

Participants who have experienced or have one or more of the following conditions will be excluded: (1) radiating pain or upper limb symptoms at areas excluding the neck (scapula included); (2) Neurological abnormalities such as decreased deep tendon reflexes, weakness and/or sensory deficits; (3) Signs or symptoms from major structural pathology such as fracture, myelopathy, neoplasm or systemic diseases; (4) History of surgery or other congenital abnormalities of the cervical spine; (5) Receiving treatment for psychiatric conditions, such as epilepsy, depressive disorder or panic disorder; (6) Suffering from pain in another area that is greater than the neck pain; (7) Received acupuncture, medication, physical therapy or manipulation therapy for neck pain within the last 2 weeks; (8) Known hypersensitive reaction after acupuncture treatment or an inability to cooperate with the acupuncture procedure; (9) Pregnant, breast-feeding or expecting a pregnancy during the study period; (10) Other factors that have been deemed inadequate for participation by research investigators.

### Randomization and Allocation Concealment

Random numbers will be generated by a computerized random number table generator through the stratified block randomization method of the SAS package (Version 9.4; SAS institute Inc., Cary, NC, USA) with a random block size. The random number table will be prepared by an independent, blinded statistician. Two block numbers will be randomly used to prevent allocation guessing by researchers. A stratified random number table will be generated according to the severity of chronic neck pain (PI-NRS score range from the 4 to 6 group vs the 7 to 10 group). The random number table file will be protected by a password, and the independent, blinded statistician, who is not involved in the recruitment, acupuncture treatment or assessment, will manage the table. A random allocation will be conducted at visit 1 to participants who provide informed consent. When a participant meets the inclusion criteria, the two types of treatment will be explained to him or her. Acupuncture will be explained as “classical acupuncture, typically used in Korean medicine clinics” and sham acupuncture as “non-classical acupuncture, rarely used in Korean medicine clinics” [14]. To ensure allocation concealment, the participants will be randomized according to the random number table by the independent researcher, and the allocated group information will be sent by an e-mail. The e-mail will be printed out and stored as a source document.

### Blinding

The participants, outcome assessor, data managers and statistician will be blinded to the allocation. The participants will be treated in the similar environments of a Korean Medicine clinic or hospital setting. All the participants will be treated with PSD regardless of the group so the participants cannot guess the allocated group by the appearance of the acupuncture. The blind will be maintained until locking of the data. To evaluate blinding, allocation guessing and a credibility rating will be assessed immediately after the first and last treatments. The practitioners and assessor are trained to treat participants according to pre-defined SOPs during the trial to maintain blindness.

### Intervention

The acupuncture treatment will be conducted by doctors of Korean medicine (DKMs) with more than 6 years of Korean medicine college education and at least 8 years of clinical experience.

To ensure the same conditions except for the needling components [15] between the groups, all the treatment procedures and regimens will be described in detail in pre-specified protocols and SOPs. A moderate interaction between the practitioners and participants is allowed: at each visit before the treatment, the practitioner will ask the participant about recent symptoms and improvements and record the answers in the patient chart; the practitioner will touch the subject’s neck to determine the pain sites according to meridian sinews; the participant will be allowed to inquire about the treatment without restriction, but the practitioner will not be allowed to give confident or positive encouragement in response to questions concerning a prognosis. This information will be given to the practitioners in workshops conducted before the study begins to ensure a standardization of the treatments. The MMRM and sham acupuncture groups will receive a total of 8 sessions (2 sessions per week) of acupuncture treatment for 4 weeks. Each acupuncture treatment will be performed after the acupuncture points are sterilized with a disposable 70 % isopropyl alcohol swab. During the treatment, the participant will lie in a prone position with a cushion under the chest to ensure a stable position.

#### Myofascial-meridian release acupuncture (MMRA) treatment

The MMRA treatment is acupuncture protocol used at the Spine and Joint Clinical Center in Kyung Hee University Korean Medicine Hospital for patients with chronic neck pain. Chronic neck pain is related to local factors such as mechanical stress from the head or spine and systemic factors such as mental stress from psychosocial issues [16, 17]. Thus, the MMRA treatment was developed to reduce not only local factors by decreasing tissue adhesion [18] and myofascial trigger points [19, 20] but also systemic factors by promoting the flow of qi and blood [21] and reducing psychological factors [22].

A total of 8 acupuncture sessions will be performed using 0.25 × 40 mm disposable sterile acupuncture needles (Dongbang Acupuncture Inc., Chungnam, Republic of Korea). As for local points, a simple insertion technique is performed at EX-B2 and 5–10 tender points on the posterior neck followed by approximately 30 s of stretching, including flexion, extension, lateral bending and rotation. The acupuncture treatment is then performed at bilateral BL10, SI15, GB20, TE15 and CV14 using PSD and real needles. The needles are retained for 20 min, and the deqi sensation is induced by rotating the needles 3–5 times in a single direction (right and left if the patient expresses pain). A maximum of 4 individual acupuncture points are allowed. For distal points, a meridian diagnosis is carried out before the acupuncture treatment. If more than 2 meridians are diagnosed, a combination of acupuncture points from both meridians can be used.

1. Greater yang meridian sinews (Smaller Intestine & Bladder meridian): BL65, SI3, Jeong-geun, Jeong-jong
2. Lesser yang meridian sinews (Triple Energizer & Gallbladder meridian): GB41, TE3, GB34, GB39
3. Yang brightness meridian sinews (Large Intestine & Stomach meridian): LI11, Young-gol, Sang-bek, CV24.

After the meridian diagnosis is complete, 6 acupuncture points in the corresponding meridian group, as well as unilateral PC6 and HT7, will be chosen for the treatment. After penetration, the needles are retained for 20 min, and the deqi sensation is induced by rotating the needles 3–5 times in a single direction (right and left if the patient expresses pain). If CV24 is chosen, the needle should be rotated right and left for 30 s after penetration, during which time flexion stretching is performed, and then removed.

#### Sham acupuncture treatment

Park sham device (PSD) will be used as sham acupuncture. It is a validated sham acupuncture device that is comprised of two tubes and a needle (either real or sham) [23]. The guide tube supports the needle when penetrating the skin vertically. A larger tube named ‘Park tube’ is attached on the ring base and lets the guide tube move along the ‘Park tube’. A silicon base is attached to the skin by a double-sided tape [13]. A placebo acupuncture needle of PSD is indistinguishable with real acupuncture, but cannot penetrate the skin. For local points, predetermined points on the posterior neck (0.5 cm from C4, 5, 6, 7 on both sides) are stimulated lightly 2 times using sham acupuncture with a blunt tip of the needle. Then, a sham acupuncture treatment is performed using the PSD and sham acupuncture needles on the points 0.5 cm away from C5 and 6 on both sides and right above the spine of scapula. The needles are retained for 20 min. For distal points, 1 cm to the inner side of both TE6 and 0.3 cm to the outer side of the highest point of both calcaneus are treated using the PSD and sham acupuncture needles. The needles are retained for 20 min without a de-qi sensation, creating a false movement that is similar to twisting acupuncture.

#### Co-interventions

The participants in both groups will not be allowed to use a pain control intervention that is related to chronic neck pain and could influence study results, such as other acupuncture treatment, medication, physical therapy, manipulation therapy, transcutaneous electrical nerve stimulation, herbal medicine, injection therapy or operation. A maximum of 3 g per day of acetaminophen, the rescue medication to relieve neck pain, will be allowed, and a dose of rescue medication will be recorded in the case report form. Brochures about good posture and head & neck exercises that are effective for neck pain relief will be offered and taught to participants on the first visit.

### Outcome measurement

The detailed outcome measurement time points are provided in Table 1.

**Table 1:** Schedule for the treatment and outcome measurements

Visit	S	1	2	3	4	5	6	7	8
Week		1		2			3	4	
Informed consent	●								
Demographic characteristics	●								
Vital signs	●	●	●	●	●	●	●	●	●
Blood test	●								●
Medical history	●								
Inclusion/Exclusion criteria	●								
Conformity assessment	●								
Treatment expectancy questionnaire	●								
Random allocation		●							
Treatment		●	●	●	●	●	●	●	●
Prescription of rescue medicine		●							
PI-NRS		●	●	●	●	●	●	●	●
NDI		●							●
CROM		●							●
HRV		●							●
Perceived stress scale		●							●
EQ-5D		●							●
PGIC									●
Pressure pain threshold		●							●
Consumption of rescue medicine									●
Days of restricted activity		●							●
Blinding test		●							●
Safety assessment		●	●	●	●	●	●	●	●

Visit 1: within 4 weeks after screening, Visit 8: 4 weeks after Visit 1 (+6 days)

*CROM* Cervical Range of Motion, *NDI* Neck Disability Index; *PGIC* Patient Global Impression of Change, *PI-NRS* 11-point Pain Intensity Numerical Rating Scale, *S* screening visit

#### Primary endpoint

The primary outcome is the mean change in the PI-NRS (0 = no pain and 10 = worst possible pain, 11-point Likert scale) from the baseline to 4 weeks. The PI-NRS has been widely used to assess chronic pain intensity in conditions such as chronic low back pain, fibromyalgia, osteoarthritis, diabetic neuropathy and post-herpetic neuralgia in placebo-controlled clinical trials [24]. In a previous study, the minimal clinically important change of PI-NRS score was 1.5 points in chronic neck pain patients [25]. Compared with global assessments of change in pain perception, the clinically important differences were a 30 % reduction of PI-NRS as “much improved” and a 50 % reduction of PI-NRS score as “very much improved,” regardless of disease, gender, age, study result or treatment group [24]. The participants will be instructed not to take a rescue medicine 4 h before assessing the pain intensity.

#### Secondary endpoint

The secondary outcomes include a change from the baseline of clinical relevance for pain (ratio of change greater than 1.5 in PI-NRS and decrease greater than 30 % and 50 % on the PI-NRS), function (NDI and Cervical Range of Motion (CROM)), autonomic and psychometric measurements (Heart Rate Variability (HRV) and Perceived Stress Scale (PSS)), quality of life (EuroQol (EQ-5D)), global assessment (Patient Global Impression of Change (PGIC)), semi-objective outcomes (pressure pain threshold (PPT), consumption of rescue medicine, days of restricted activity), immunologic and stress markers and adverse events.

##### Pain

The mean change of pain intensity from baseline at 1, 2, 3 weeks will be measured by the PI-NRS. Clinical relevance will be assessed using the ratio of participants in each group who reported PI-NRS scores that reduced greater than 1.5 points or 30 % and 50 % at 4 weeks from the baseline PI-NRS scores. We pre-defined the type of improvements as follows: a PI-NRS reduction of 1.5 points as minimally important; 30 % reduction from baseline as moderately important; and 50 % reduction as substantially important [26]. Therefore, in this trial, both the absolute and relative score change will be used to analyze the clinical relevance.

##### Disability

The function change in the neck will be evaluated using the Neck Disability Index (NDI) and cervical range of motion (CROM) from baseline to 4 weeks. The NDI is the most frequently used questionnaire to evaluate cervical pain and function limitation in daily life and consists of 10 questions ranging from 0 to 50 points. A higher score indicates more severe symptoms. The reliability and validity of the Korean version of the NDI was examined in 2009 by Song et al. [27]. The CROM of flexion, extension, lateral bending and rotation will be measured by a CROM device (Performance Attainment Associates, Lindstrom, MN, USA). A previous systematic review showed that the reliability and validity of the CROM device were favorable compared with other methods for assessing CROM [28].

##### Autonomic and psychometric measurement

The mean change from baseline for autonomic and psychometric measurements will be assessed by heart rate variability (HRV) and perceived stress scale (PSS). HRV will be evaluated by the commercial device PowerLab Biomap ML132 (AD Instrument, Castle Hill, Australia) with a 400 Hz sampling rate. HRV measurements will be conducted according to the guidelines written by the task force of the European Society of Cardiology and the North American Society of Pacing and Electrophysiology [29]. The standard deviation (SD) of the NN interval, square root of the mean squared differences of successive NN intervals, Low frequency (LF), High frequency (HF), LF/HF ratio and Total Power will be evaluated. Developed in 1983 by Cohen [30], the PSS is a self-reported questionnaire that evaluates degree of the individual’s perceived stress with a 5 point-Likert scale consisting of 14 questions. The questionnaire was translated into Korean, and the reliability and validity of the Korean version of the PSS [31] was studied.

##### Quality of life

EQ-5D will be used to determine the quality of life of a patient with chronic neck pain. The mean change from the EQ-5D-5L baseline will be used for analysis. The EQ-5D is a standardized instrument to measure health outcomes that offers generic questions about quality of life as it relates to personal health status. We will use the Korean version of the EQ-5D [32].

##### Global assessment

The participants’ global assessment will be performed using the PGIC, a self-reported 7-point categorical scale used to evaluate the overall improvement after treatment [24]. Participants will be asked to evaluate themselves subjectively regarding the improvement of their symptoms at the 4th week compared to baseline by selecting one of the following seven options: 1) very much improved, 2) much improved, 3) minimally improved, 4) no change, 5) minimally worse, 6) much worse or 7) very much worse.

##### Semi-objective outcomes

Semi-objective outcomes, such as the PPT with a pressure algometer, consumption of rescue medicines and days of restricted activity, will be checked. PPTs will assess the bilateral levator scapulae, trapezius descendens and paravertebral of the 6th cervical spine [33] using a hand-held dial Pressure Algometer Device (Baseline Push Pull Force Gauge Model 12–0304 (Fabrication Enterprises Inc., New York, USA)). We will check three times at each point and use the mean value (kg/cm2). This device has good inter-tester reliability even when performed by inexperienced testers [34]. The rescue medication, a maximum 3 g of acetaminophen per day, will be allowed, and the dose between two groups will be assessed at 4 weeks. Days of restricted activity are known to be a semi-objective outcome that can evaluate subjective symptoms through digitization [35]. In this study we have modified the “restricted activity page” from “Current estimates from the National Health Interview Survey, United States, 1988” questionnaire to suit chronic neck pain patients. The participants will be asked to answer the following three questions: 1) “How many days during the last 2 weeks were you forced to spend more than a quarter of a day (approximately 2–3 h) lying down due to neck pain?” 2) “How many days during the last 2 weeks were you forced to miss work or school for more than a quarter of a day (approximately 2–3 h) due to neck pain?” 3) How many days during the last 2 weeks did you feel that daily activities were restricted for more than a quarter of a day (approximately 2–3 h) due to neck pain?” (exclude days answered in questions 1 & 2)

##### Biomarkers

Inflammatory/immunologic and stress biomarkers will be assessed at 1 and 4 weeks after randomization. Biomarkers of inflammation such as ESR and CRP; immunologic markers such as IL-1B, IL-6, TNF-α, IFN-γ; and stress markers such as serum cortisol were chosen according to previous research [36, 37]. Blood samples were collected from 10:00 to 11:30 am to minimalize the cortisol changes throughout the day.

#### Safety and adverse events outcomes

All unexpected and unintended responses that are not necessarily related to the acupuncture treatment will be recorded at every visit on an adverse event report form by the practitioners and the participant. Adverse events known to be related to acupuncture treatments include pain, bruising, bleeding, dizziness, anxiety and infection [38]. A causal relationship between the acupuncture treatment and adverse events will be evaluated using a 6-grade scale (1 = definitely related, 2 = probably related, 3 = possibly related, 4 = probably not related, 5 = definitely not related and 6 = unknown), and the seriousness of the adverse events will be scored using a 4-point scale (1 = mild, 2 = moderate, 3 = severe and 4 = extremely severe).

#### Blinding assessment

The allocation guessing about real and sham acupuncture treatments will be assessed after the first treatment and at the end of the treatment. The participants will select questionnaires on their feelings about the acupuncture treatments using the following three questions: “classical acupuncture typically used in Korean medicine clinics”, “non-classical acupuncture, rarely used in Korean medicine clinics” or “don’t know”. The Credibility rating for both treatments will be evaluated using a credibility test with a 7-point Likert scale developed by Vincent et al. after their first treatment and at the end of the treatment [39]. The participants will rate the scores (0 = very low confidence and 6 = very high confidence) for the following four questions, “How confident do you feel that this treatment can alleviate your chronic neck pain?”; “How confident would you be in recommending this treatment to a friend who suffered from a similar complaint?”; “How logical does this treatment seem to you?”; “In your opinion how successful may this treatment be in alleviating other complaints?”

### Sample size calculation

The primary outcome of our study is overall neck pain, which is measured by the PI-NRS change from baseline to 4 weeks. In chronic neck pain, the minimum clinically important difference (MCID) is a 1.5 unit change in the PI-NRS score [25]. According to a previous study [40], we assumed that the SD of PI-NRS change from baseline to 4 weeks will be 1.88 in the treatment group and 2.21 in the control group, each with a population correlation between the baseline and follow-up scores that was estimated as 0.5. In a superiority test, we assumed that the PI-NRS difference between the two groups will be 1.5, which is identical to the MCID. With a 5 % of significance level (α = 0.05, 2-sided) and 80 % power (1-ß = 0.8), the sample size was calculated as 31 participants in each group using G*Power 3.13 software (Franz Faul, Universität Kiel, Germany). Considering a 15 % drop-out rate, a total of 74 participants is needed.

### Statistical analysis

The analysis set will consist of a full analysis set (FAS), per protocol (PP) set and safety set. The safety set will include any participants who were randomly assigned and received at least one acupuncture treatment. The FAS will include data indicating that the treatment was as close to intention to treat (ITT) as possible. In addition to the criteria of the safety set, to be included in the FAS participants have to be evaluated for the primary outcome at least once. To be included in the PP set, participants have to receive more than 6 sessions (75 %) of treatment. The FAS will be used in the main analysis. The primary outcome of our research is the difference in PI-NRS score change from the baseline to week 4 between the two groups. Sensitivity analysis will be performed by comparing the results from PP analysis and ITT analysis, and the last observation carried forward (LOCF) method will be used to handle the missing data.

For the descriptive analysis, data will be evaluated for normality and log-transformed when necessary. A two student’s t-test or a Wilcoxon rank sum test for continuous data and a chi-squared test or Fisher’s exact test for categorical data will be performed according to whether the data are normally distributed or skewed.

For a confirmatory analysis, primary efficacy endpoint, the change from baseline to week 4 in PI-NRS, will be evaluated using student’s t-test or Wilcoxon rank sum test. This result will be compared with adjusted result using an analysis of covariance (ANCOVA) with the baseline measurement of PI-NRS as a covariate, the treatment group as a fixed effect. The secondary efficacy endpoint will be evaluated using a student’s t-test or Wilcoxon rank sum test for continuous data or chi-squared test or Fisher’s exact test for categorical data. All of the statistical analyses will be performed SAS package (Version 9.4; SAS institute Inc., Cary, NC, USA) by a statistician blinded to patient allocation, and a significance level of 5 % will be used.

### Data and safety monitoring

To ensure the quality of the data according to the pre-determined protocol and SOPs, regular monitoring will be conducted. Monitors will be blinded to the allocation and will evaluate if the recruitment procedures and data recording followed the protocol in the case report forms. If there is a need for modifications in the study methods, such as changes to the eligibility criteria, treatment regimens or duration of follow-up, the principal investigator may discuss the matter with independent researchers and statisticians. Should severe adverse events or crucial issues occur, the principal investigator will determine whether the issues are acceptable or whether it is necessary to change or terminate the trial.

### Participant protections and ethics

The study was planned in accordance with the Helsinki Declaration and the Korean Good Clinical Practice Guidelines to protect the participants and was approved by the institutional review board (IRB) of Kyung Hee University Korean Medicine Hospital (KOMCIRB-150217-HRBR-010). The participants will be informed about the potential benefits, risks, alternatives and responsibilities during the study by the researchers throughout the consent process, and the written informed consent will be obtained from each participant. To avoid potential adverse events, in case a practitioner considers a participant unsuitable for acupuncture treatment due to an abnormal health condition such as moderate fatigue or a common cold, the treatment will be rescheduled within 3 days.

## Discussion

The purpose of our study is to evaluate the efficacy of an MMRA treatment in chronic neck pain patients. Considering the limitations mentioned in a previous systematic review [9, 41], we will assess semi-objective outcomes and laboratory biomarkers in addition to subjective outcomes. Clinical relevance and statistical significance were also considered when calculating the sample size and interpreting the results [25]. Moreover, the MMRA treatment that is actually used for chronic neck pain at Spine and Joint Clinical Center in Kyung Hee University Korean Medicine Hospital was reflected in the protocol to increase the external validity. The safety of the MMRA treatment will also be evaluated.

Specifically, we will focus on the mechanism of the acupuncture treatment in chronic neck pain. Biomarkers of inflammation, such as IL-1β, IL-6, CRP and TNF- α, are known to be related to musculoskeletal pain [7, 36, 37]. Several animal studies showed that acupuncture stimulation changed cytokine levels by endogenous opioid and non-opioid means [42]. Electroacupuncture treatments are also known to reduce pro-inflammatory cytokines in peripheral lesions and the spinal cord [43]. Recently, electroacupuncture has been suggested to have an anti-inflammatory effect and affect the production of catecholamines in the adrenal glands by mediating the sciatic and vagus nerves [44]. Various stress variables, such as cortisol level, a stress questionnaire and HRV, are also likely to be correlated with neck pain [8, 45–48]. In an acupuncture trial in knee osteoarthritis, 10 days of an electroacupuncture treatment resulted in a significant improvement in objective measures of stress-associated biomarkers, such as plasma cortisol, as well as subjective measures, such as pain, stiffness and disability. In another acupuncture trial in chronic fatigue syndrome, 10 sessions of acupuncture treatment for 4 weeks reduced the stress measured by a Stress Response Inventory questionnaire. In a pilot study with night-shift-working nurses, acupuncture treatment attenuated the imbalance between sympathetic and parasympathetic activities measured by HRV [49]. However, whether acupuncture treatment reduces chronic neck pain through inflammation and stress levels is not clear. In this study, we will conduct an exploratory data analysis to elucidate the role of MMRA treatment in managing inflammation and stress levels in chronic neck pain patients to determine if acupuncture is associated with physiological changes beyond the placebo effects.

To summarize, our protocol adopted several improvements to overcome the limitations of previous chronic neck pain acupuncture research while considering the clinical relevance and determining a mechanism of acupuncture treatment.

### Trial status

Recruitment will begin in November 2015 and will be completed by the end of April 2016. We expect the results will be reported by the end of 2016.

## Abbreviation

ANCOVA: Analysis of covariance
CROM: Cervical range of motion
DKMs: Doctors of Korean medicine
EQ-5D: EuroQol
FAS: Full analysis set
HF: High frequency
HRQoL: Health-related quality of life
HRV: Heart rate variability
ITT: Intention to treat
LF: Low frequency
LOCF: Last observation carried forward
MCID: Minimal clinical important difference
MMRA: Myofascial-meridian release acupuncture
NDI: Neck Disability Index
PGIC: Patient global impression of change
PI-NRS: the 11-point pain intensity numerical rating scale
PP: Per protocol
PPT: Pressure pain threshold
PSD: Park sham device
PSS: Perceived stress scale
SD: Standard deviation
TNF-α: Tumor necrosis factor- α
VAS: Visual analogue scale
